# Extracellular nitric oxide sustains root surface redox activity and growth under sudden flooding-induced hypoxic conditions in barley root tips

**DOI:** 10.1007/s00425-023-04279-w

**Published:** 2023-11-21

**Authors:** Veronika Zelinová, Loriana Demecsová, Ľubica Liptáková, Katarína Valentovičová, Ladislav Tamás

**Affiliations:** Institute of Botany, Plant Science and Biodiversity Centre, Slovak Academy of Sciences, Dúbravská cesta 9, SK-84523 Bratislava, Slovak Republic

**Keywords:** Cell death, Lipid peroxidation, Root growth inhibition, Trans-plasma membrane electron transport

## Abstract

**Main conclusion:**

Nitric oxide sustains root tip surface redox activity and restricts lipid peroxidation-triggered cell death in the root tips.

**Abstract:**

In order to gain more insight into the involvement of nitric oxide (NO) in plant response to sudden flooding-induced hypoxic stress, we studied the effect of two NO donors, sodium nitroprusside and S-nitroso-L-glutathione, on short-term partial submergence-induced root growth inhibition, alteration in root surface redox activity, lipid peroxidation and cell death in two barley cultivars (cv.) at their early seedling stage. The short-term hypoxic stress induces root growth arrest in cv. Karmel, accompanied by increased lipid peroxidation and cell death. By contrast, in cv. Slaven, short-term hypoxic conditions cause only reduced root growth rate, associated with elevated extracellular NO level in the root tips. The root tip surface redox activity decreases with the increasing timespan of hypoxic conditions in both cultivars; however, this decrease in redox activity started earlier and was greater in the cv. Karmel in comparison with cv. Slaven. Application of NO donors during hypoxic stress sustains the root redox activity and eliminates the hypoxia-induced lipid peroxidation, accompanied by a partial restoration of root growth after short-term hypoxic stress. These results suggest that extracellular NO plays a key role in maintaining the root tip surface redox activity and in the restriction of lipid peroxidation and cell death under short-term hypoxic stress in the root tips of barley seedlings.

**Supplementary Information:**

The online version contains supplementary material available at 10.1007/s00425-023-04279-w.

## Introduction

Due to the growth of human population, agricultural productivity will have to increase, while maintaining food security. However, plants, especially agriculturally important crops, face adverse challenges, such as stress caused by drought, heavy metals, flooding, etc., which affects their growth and yield. Climate projection models made by Hirabayashi et al. (2013) suggest an increase in frequency and severity of flooding events in major farming regions. Another estimate supposes that the losses sustained through floods will increase up to five folds (Alfieri et al. 2016). In the case of barley production, high rainfall can reduce its yield up to 50% (Setter and Waters 2003).

Plant flooding can vary in nature from waterlogging for a short period (a few hours) to complete submergence during a prolonged period (weeks and months). However, all types of flooding limit plant growth and development by the alteration of numerous morphological, physiological and biochemical processes; in very sensitive plants, they may even evoke cell death within a few hours (Bailey-Serres and Voesenek 2008; Bailey-Serres et al. 2012; Fukao et al. 2019). Tissue oxygen deficiency is the very early symptom of seedlings under flooding, as a consequence of a marked reduction in gas diffusion in water resulting in hypoxic conditions for the flooded parts of seedlings (Bailey-Serres et al. 2012). Therefore, the characteristic morpho-anatomical adaptation response of waterlogged plants, including barley, is the ability to form new adventitious roots with a large volume of aerenchyma (Sauter 2013; Zhang et al. 2015). Furthermore, one of the most common features of all submerged plant tissues is a shift from aerobic respiration to anaerobic metabolism to sustain ATP production (Bailey-Serres and Voesenek 2008). Accordingly, the activity of fermentative enzymes and the accumulation of lactate and ethanol in the root tissues were detectable within a few hours of flooding (Da-Silva and do Amarante 2020a).

The root tip is the most sensitive root part to hypoxic conditions under flooding, resulting in extensive cell death within a short time period (Gladish et al. 2006; Cheng et al. 2013). Within the root tip, the most sensitive part to sudden lack of oxygen is the root transition zone, and as such plays essential roles in both sensing and adapting to hypoxic conditions (Mugnai et al. 2012). Although, under flooding conditions, the availability of oxygen is markedly reduced, in several plant species, enhanced reactive oxygen species (ROS) generation has been observed under flooding stress and particularly during the post-flooding reoxygenation (Blokhina et al. 2003). Increasing evidence indicated that ROS are involved in both signaling and cell damage under hypoxic conditions (Santosa et al. 2007; Pucciariello and Perata 2017; Sasidharan et al. 2021).

One of the characteristic features of both animal and plant cells under hypoxic conditions is the considerable increase in nitric oxide (NO) production (Dordas et al. 2003; Feelisch 2018). The role of NO under flooding-induced hypoxic conditions is well documented in plants, including barley (De Castro et al. 2022). It enables the consumption of NADH and the production of a small amount of ATP via the phytoglobin-NO cycle as an alternative respiration pathway, which is indispensable to survive under flooding (Igamberdiev et al. 2005; Gupta et al. 2020). In addition, this mechanism functions also during seed germination reducing high oxygen consumption, so the mitochondrial respiration is adjusted, and the toxic hypoxic conditions in tissues are prevented (Benamar et al. 2008). Moreover, NO regulates respiration and the balance of oxygen and ROS in root mitochondria under aerobic conditions (Gupta et al. 2014). Besides its role in the phytoglobin-NO cycle, NO is via enhanced expression and capacity of alternative oxidase also involved in the activation of the alternative respiration pathway, leading to a suppression of mitochondrial ROS generation (Kumari et al. 2022). On the other hand, enhanced phytoglobins-mediated NO depletion and consequent stabilization of ethylene-responsive transcription factors initiate the adaptation responses to hypoxic conditions during the submergence of plant tissues (Mira et al. 2016; Hartman et al. 2019). In addition to these intracellular functions, several possible roles of NO have been reported in the apoplastic space of root tissues under both physiological and stress conditions (Stöhr and Ullrich 2002).

In this study, we analyzed the role of ROS and NO in partial submergence-caused short-term hypoxic stress-induced root growth inhibition, alteration in root surface redox activity, antioxidant enzyme activities, superoxide generation, lipid peroxidation and cell death in two barley cultivars (cvs.), Karmel and Slaven, at their early seedling stage. In addition, in order to gain more insight into the involvement of NO in these responses, we studied the effect of two NO donors, sodium nitroprusside (SNP) and S-nitroso-L-glutathione (GSNO), and an NO scavenger, 2-(4-carboxyphenyl)-4,4,5,5-tetramethylimidazoline-1-oxyl-3-oxide (cPTIO), on partially submerged roots. The potential side effect of cyanide release during SNP treatment was checked by the use of potassium ferricyanide (PFiC).

## Material and methods

### Plant material and growth conditions

Seeds of two barley (*Hordeum vulgare* L.) cvs., Karmel and Slaven (Plant Breeding Station, Hordeum Ltd, Sládkovičovo-Nový Dvor, Slovakia), were briefly (1-2 min) rinsed in distilled water and then germinated at the temperature of 22 ± 2 °C in dark, between wetted filter paper for 20 h. Well-germinated seeds with a visible protrusion of a radicle were arranged into rows between two sheets of moist filter paper on rectangle trays. Afterwards, these trays were placed in a nearly vertical position (so the root growth is guided downwards) and were moisturized by using a wick of filter paper soaked in a reservoir of distilled water. Three days after germination and incubation in the dark at 22 ± 2 °C, when primary root length reached approximately 4–5 cm, seedlings were selected for the experiments. Short-term hypoxia stress was imitated by the immersion of 30 seedlings (partial submergence the upper part of seedlings was not submerged) into 100 mL of distilled water or into different concentrations (see figure legends) of SNP, PFiC, GSNO, cPTIO or warfarin for 0.5, 1, 2 or 3 h. The GSNO (1 mM) was synthetized by mixing equal volumes of GSH (2 mM) and NaNO_2_ (2 mM) prepared in 0.1 N HCl. After stirring the reaction mixture for 10 min, the pH was adjusted to 6.0 by NaOH. Dissolved oxygen concentration was measured, using a dissolved oxygen meter, either without or with the different number of seedlings 1, 2 and 3 h after the immersion of seedlings (roots) into 100 mL of distilled water.

### Post-hypoxic root growth analysis

To determine the post-hypoxic root growth increment, after 1, 2 or 3 h of hypoxic conditions, the seedlings were incubated for 6 h in a vertically oriented tray between two sheets of filter paper, as described above. The position of each seedling's longest root tip was marked on the filter paper at the beginning of incubation. Following the 6 h of incubation, the increment in root length was measured by an image analyzer after the root tips were excised at the marked place on the filter paper. The values are the means of five independent experiments (30 roots per experiment).

### NO detection and localization

Three millimeters-long apical segments of each seedling's two longest roots (15 segments per reaction) were washed (5 min) in 20 mM sodium phosphate buffer, pH 6.0; before their incubation (for 30 min at 30 °C, with shaking) in a 400 µL of reaction mixture containing: 20 mM sodium phosphate buffer (pH 6.0), 5 µM 4-amino-5-methylamino- 2′,7′-difluorofluorescein (DAF-FM, from 5 mM DMSO stock solution) and 1 mM potassium cyanide (KCN). KCN was used in the reaction mixture to inhibit a metabolic decomposition of NO by peroxidases and oxidoreductases in the root apoplast. NO scavenger, cPTIO, at 0.1, 0.25, 0.5 and 1 mM concentrations was added into the reaction mixture to control the NO specificity of the reaction. After the removal of root segments, the fluorescence intensity was measured in 300 µL of the reaction mixture using a microplate reader at Ex/Em: 495 (filter 485/20)/515 (filter 528/20) nm. The amount of NO generated by root segments was expressed in a relative fluorescence unit (RFU), after the background (reaction mixture without root segments) fluorescence was subtracted.

For NO localization, intact seedlings incubated (in darkness for 45 min at 25 °C) in 20 mM sodium phosphate buffer, pH 6.0, containing 1 mM potassium cyanide and 5 μM diaminorhodamine-4M acetoxymethyl ester (DAR-4M-AM; from 5 mM stock in DMSO) were used. After washing the roots for 5 min with distilled water, root fluorescence intensity (two longest roots of each seedling) was detected by a fluorescence stereomicroscope (Ex/Em: 545 ± 25/606 ± 70 nm). The presented images are the representative of five independent experiments. The staining and fluorescence intensity (grey values) were analyzed by the ImageJ software.

### Superoxide localization

Superoxide localization was studied in intact roots using hydroethidine (HE), which detects mainly superoxide generated in the mitochondria and nitro-blue tetrazolium chloride (NBT), which in the presence of azide reacts mainly with apoplastic superoxide generated by the diphenyleneiodonium (DPI)-sensitive NADPH oxidases, according to Valentovičová et al. (2022).

### Lipid peroxidation assay

Lipid peroxidation was determined by measuring the amount of thiobarbituric acid reactive substance (TBARS). Apical segments (3 millimeters long) of the two longest roots of each seedling (30 segments per reaction) were excised and placed into 500 µL of 10% trichloroacetic acid containing 0.5% thiobarbituric acid and homogenized using a cold pestle and mortar. After incubation for 15 min at 95 °C, the homogenate was quickly cooled on ice and centrifuged at 12 000 *g* for 10 min in a cold rotor. The absorbance of the supernatant (300 µL) was measured at 532 nm and at 600 nm for correction.

### Cell viability assay

The loss of cell viability was evaluated using the Evans blue staining method. Intact roots after treatments were stained with 0.025% (v/v) aqueous solution of Evans blue for 45 min. After washing with distilled water (3 × 5 min), three millimeters long apical segments of each seedling's two longest roots (20 segments per reaction) were excised and homogenized in the extraction solution containing 50% methanol and 1% SDS. Following incubation (1 h at 70 °C), extracts were centrifuged for 10 min at 12 000 *g*. The optical density of released Evans blue was measured in the supernatant (300 µL) spectrophotometrically at 600 nm.

### Root surface redox activity

Root surface redox activity was estimated using a cell-impermeable tetrazolium salt, XTT (sodium 3′-[1-[(phenylamino)-carbonyl]-3,4-tetrazolium]-bis(4-methoxy-6-nitro)benzene-sulfonic acid hydrate), in conjunction with an intermediate electron acceptor phenazine methosulfate (PMS), according to Cory et al. (1991) with some modifications. Twenty, three millimeters long, apical segments of the two longest roots of each seedling were cut, followed by incubation in 400 µL of distilled water for 5 min. Then, the washing distilled water was changed for the 400 µL of reaction mixture containing 0.5 mM XTT and 0.1 mM PMS, and the samples were incubated in the dark with gentle shaking at 30 °C for 30 min. The effect of NO scavenger on root surface activity was analyzed by the addition of cPTIO into the reaction mixture. The absorbance of the reaction mixture (300 µL) without root segments was recorded at 470 nm. Root surface activities were expressed as an increase in absorbance per root segment and minute (A_470_ root segment^−1^ . min^−1^).

### Enzyme activity assays

For protein extraction, apical segments (3 millimeters long) of the two longest roots of each seedling (30 segments per extraction) were homogenized in a pre-cooled mortar with 100 mM potassium phosphate extraction buffer, pH 7.8, containing 1 mM EDTA. After centrifugation at 12 000 *g* for 10 min, proteins were quantified with bovine serum albumin as the calibration standard by the method of Bradford (1976).

Glutathione peroxidase (GPX, EC 1.11.1.9) activity was measured according to Drotar et al. (1985) with some modifications using a microplate reader (Synergy HT BIO-TEK, USA). The reaction mixture (200 μL) contained 2 mM glutathione, 0.5 mM NADPH, 1 mM EDTA, 2 mM t-butyl hydroperoxide and 0.5 U of glutathione reductase in 100 mM sodium phosphate buffer, pH 7.0, and 30 μg of extracted proteins. The rate of NADPH oxidation was measured at 340 nm over a time period of 20 min at 30 °C. Specific GPX activities were expressed as ΔA_340_ mg^−1^min^−1^.

Glutathione reductase (GR, EC 1.6.4.2) activity was determined by measuring the increase in absorbance at 412 nm, reflecting the reduction of 5,5′-dithiobis (2-nitrobenzoic acid) by reduced glutathione (Smith et al. 1988). The reaction mixture contained 50 mM potassium phosphate buffer, pH 7.8, 0.5 mM EDTA, 0.5 mM oxidized glutathione, 1 mM DTNB, 0.25 mM NADPH and 10 μg of proteins from root extract. The reaction mixture (200 μL) was incubated at 30 °C for 20 min. Specific activities were expressed as ΔA_412_ mg^−1^min^−1^.

### Statistical analyses

Each value is the mean of five independent experiments with three replicates per experiment. The data were analyzed by one-way analysis of variance (ANOVA test), and the means were separated using Tukey’s test.

## Results

### Hypoxia tolerance is associated with enhanced NO level, while sensitivity with lipid peroxidation and cell death in the root tip

In our experimental conditions, the dissolved oxygen concentration in distilled water was 4.5 ± 0.31 mg/L; however, its level rapidly decreased in the presence of roots in a seedling number- and time-dependent manner, reaching nearly zero (0.6 ± 0.16 mg/L) concentration 3 h after the immersion of 30 seedlings into 100 mL of distilled water (Table 1). Moreover, immersion of 30 barley seedlings with primary roots length approximately 4–5 cm into 100 mL of distilled water, without oxygen replenishment, evoked hypoxic stress (oxygen concentration below 3 mg/L) within an hour.

**Table 1:** Effect of barley (cv. Karmel) seedling number and submergence time on dissolved oxygen concentration (mg/L) in 100 mL of distilled water (dissolved oxygen concentration in distilled water without seedlings 4.5 ± 0.31 mg/L). Mean values ± SD (*n* = 5)

Number of seedlings/100 mL DW	Timespan of submergence 1, 2, 3 h Dissolved oxygen concentration (mg/L)		
**5**	4.5 ± 0.35	3.6 ± 0.2	3.2 ± 0.3
**10**	4 ± 0.46	3.1 ± 0.3	2.3 ± 0.38
**20**	2.8 ± 0.4	1.7 ± 0.42	1 ± 0.2
**30**	2.1 ± 0.36	0.9 ± 0.25	0.6 ± 0.16

**Fig. 1:**
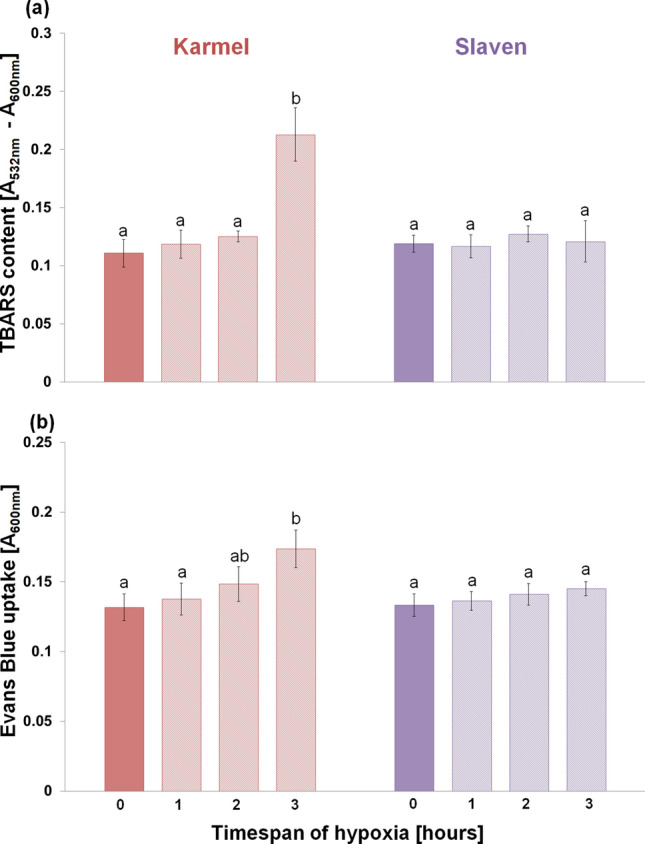
Lipid peroxidation (**a**) and cell death (**b**) analysis in the root tips of barley cvs. Karmel and Slaven after 0 (normoxia), 1, 2 and 3 h of hypoxic stress. Mean values ± SD (*n* = 5). Different letters indicate statistical significance according to Tukey’s test (*P < 0.05*)

**Fig. 2:**
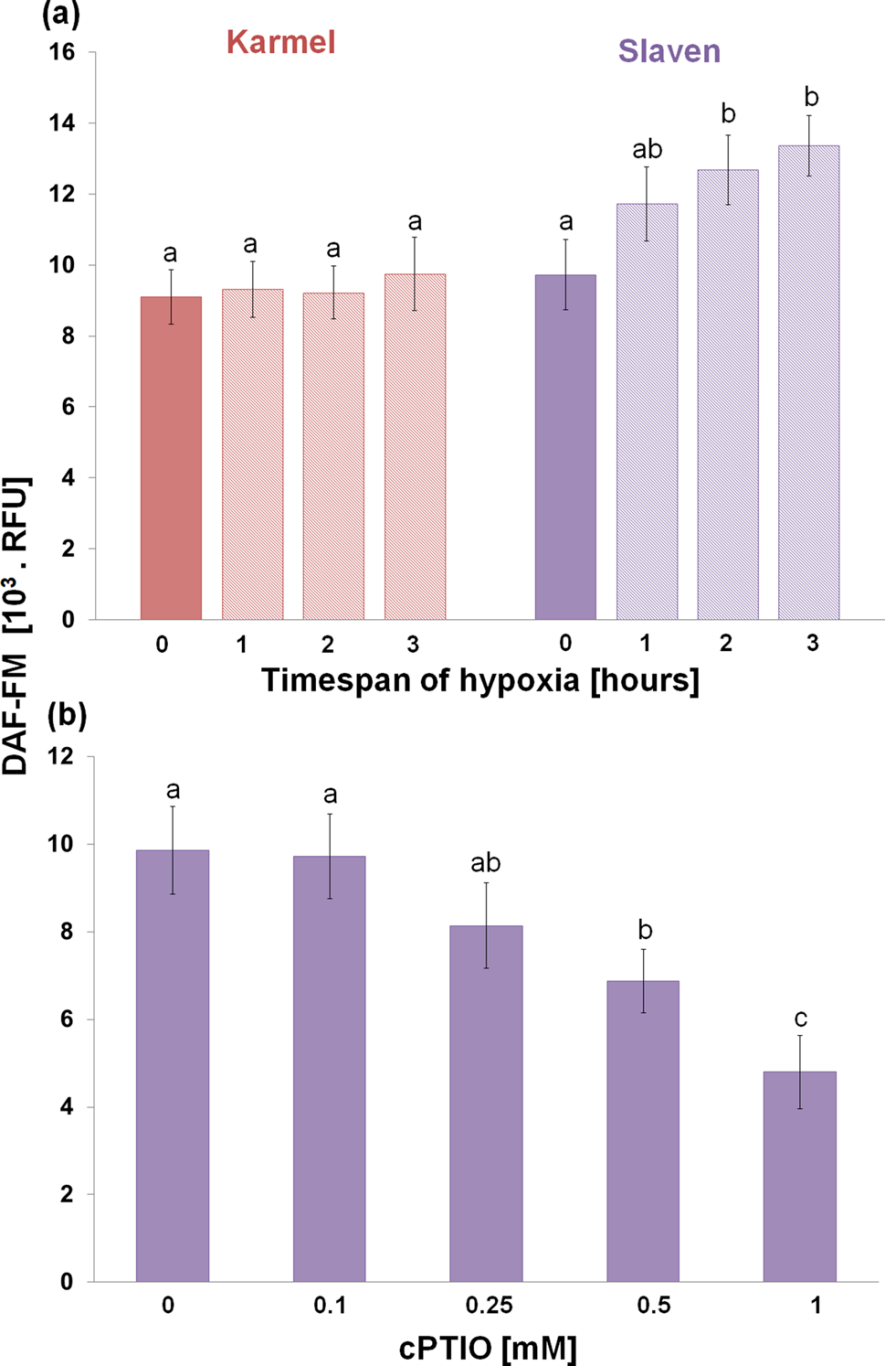
NO release from the root tips of barley cvs. Karmel and Slaven after 0 (normoxia), 1, 2 and 3 h of hypoxic stress (**a**) and the effect of cPTIO in the reaction mixture on the level of NO release from the normoxic root tips of cv. Slaven (**b)**. Mean values ± SD (*n* = 5). Different letters indicate statistical significance according to Tukey’s test (*P < 0.05*)

**Fig. 3:**
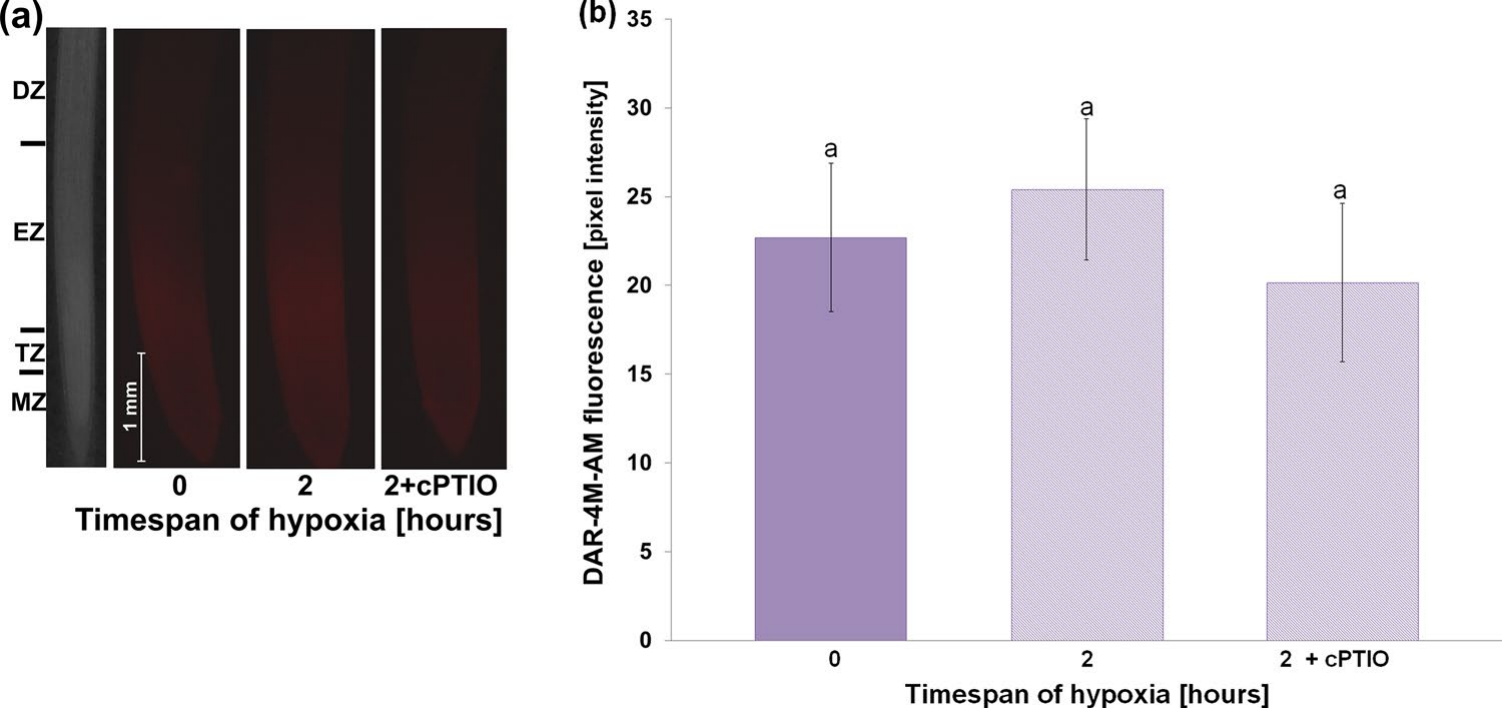
NO localization (**a**) and intensity of DAR-4M-AM fluorescence (**b**) in the root tips of barley cv. Slaven after 0 (normoxia) and 2 h of hypoxic stress without and with 1 mM cPTIO in the staining solution. Mean values ± SD (*n* = 5). Different letters indicate statistical significance according to Tukey’s test (*P < 0.05*)

In cv. Karmel, 3 h of hypoxic stress significantly increased the level of lipid peroxidation, which resulted in extensive cell death (Fig. 1). On the contrary, such an increase in lipid peroxidation and cell death was not observed in cv. Slaven. On the other hand, the amount of released NO by the root tips increased under hypoxic conditions only in the root tips of cv. Slaven (Fig. 2a). In the presence of NO scavenger, cPTIO, in the reaction mixture, the amount of DAF-FM-detectable extracellular NO decreased in a cPTIO concentration-dependent manner (Fig. 2b). KCN was applied into the reaction mixture to avoid the peroxidases and oxidoreductases-caused extracellular NO depletion. As both peroxidases and dioxygenases-catalyzed reaction are very sensitive to KCN, the presence of KCN markedly increased the amount of DAF-FM-detectable extracellular NO released by the root apical segments (Suppl. Fig. S1a). This increase started at 0.01 mM KCN concentration and reached the maximum level at 1 mM concentration, while higher KCN concentrations lowered the DAF-FM fluorescence signal in comparison with 1 mM concentration. The amount of released NO increased with increasing number of root tips in the reaction mixture nearly linearly (up to 30 root tips per reaction) in the presence of KCN, whereas without KCN, it was hardly detectable (Suppl. Fig. S1b). At the same time, KCN alone had no effect on the background (reaction mixture without root segments) fluorescence intensity. In contrast to enhanced extracellular NO generation during hypoxic stress, the analysis of NO localization did not show significant changes in intracellular NO levels in the root transition and elongation zone under hypoxic stress (Fig. 3). Furthermore, cPTIO did not decrease significantly the intensity of DAR-4M-AM fluorescence in the root tips (Fig. 3).

Despite the high degree of lipid peroxidation in cv. Karmel, the signal for HE-detectable superoxide did not change significantly with increasing hypoxic stress (Suppl. Fig. S2). Moreover, the intensity of DPI-sensitive NBT staining for superoxide decreased rapidly in roots of cv. Karmel even after 2 h of hypoxic stress (Suppl. Fig. S3). No significant changes were observed in the activity of GPX and GR under short-term hypoxic stress in cvs. Karmel or Slaven (Suppl. Fig. S4).

### Hypoxia and NO scavenger reduce root surface redox activity

**Fig. 4:**
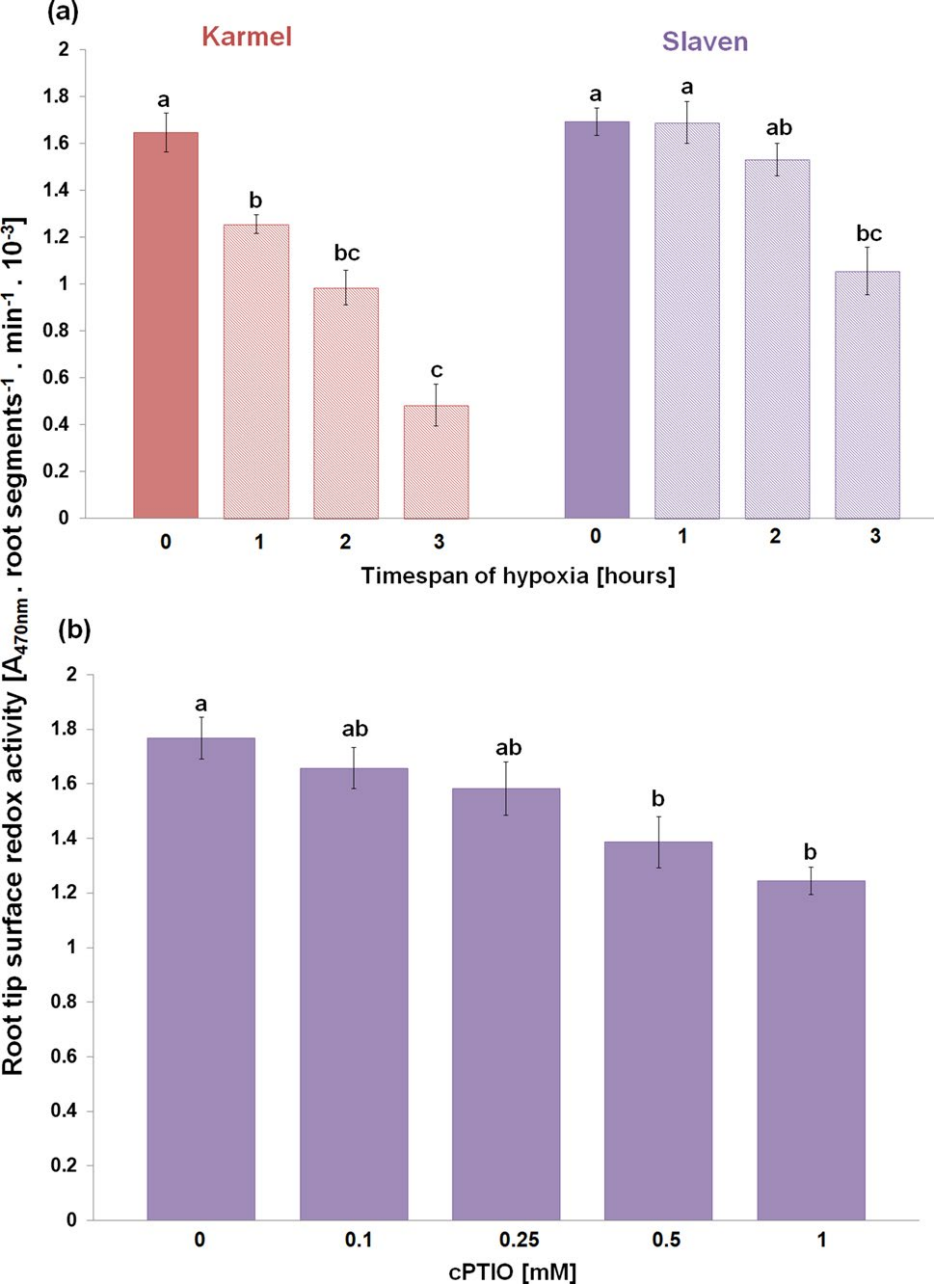
Root tip surface redox activity of barley cvs. Karmel and Slaven after 0 (normoxia), 1, 2 and 3 h of hypoxic stress (**a**) and the effect cPTIO in the reaction mixture on root surface redox activity in normoxic root tip of barley cv. Slaven (**b**). Mean values ± SD (*n* = 5). Different letters indicate statistical significance according to Tukey’s test (*P < 0.05*)

The root tip surface redox activity decreased with the increasing timespan of flooding-induced hypoxia in both cvs. (Fig. 4a); however, the cv. Karmel had a greater decrease in redox activity than cv. Slaven. In addition, this decrease in cv. Karmel was significant even after 1 h of hypoxic conditions. Application of NO scavenger, cPTIO, into the reaction mixture decreased the surface redox activity of the unstressed/normoxic root tips within 30 min of treatment (Fig. 4b). While at low cPTIO concentrations (0.1–0.25 mM), the surface redox activity of root tips was only a little affected, at higher concentrations, significantly lower activity was observed in comparison with the control without cPTIO.

### NO donors restore root surface redox activity and growth and eliminate hypoxia-induced lipid peroxidation

**Fig. 5:**
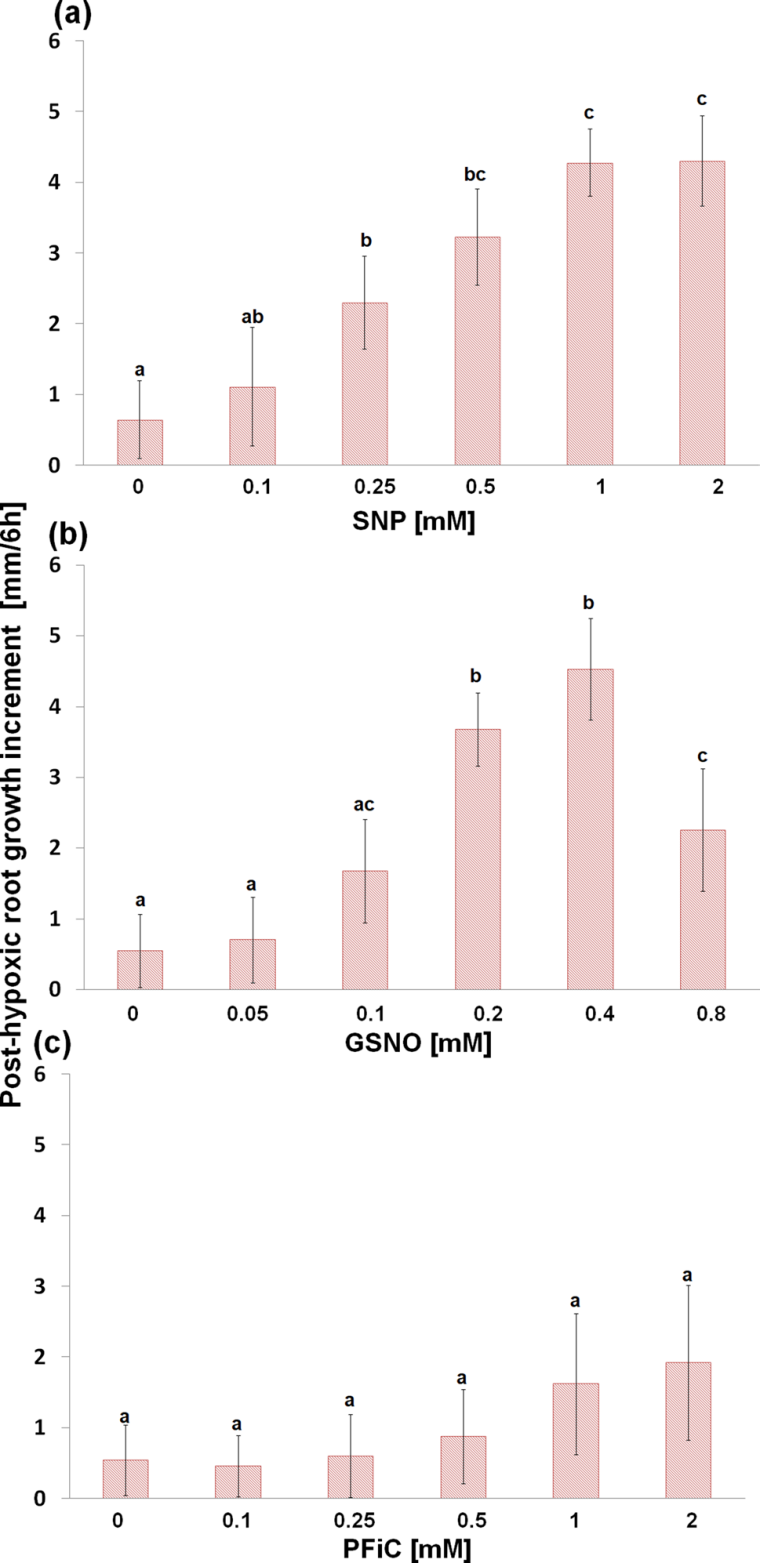
Effect of SNP (**a**), GSNO (**b**) and PFiC (**c**) during 3 h of hypoxia on the post-hypoxic root length increment during 6 h of recovery of barley cv. Karmel. Mean values ± SD (*n* = 5). Different letters indicate statistical significance according to Tukey’s test (*P < 0.05*)

Application of NO donor, SNP, during hypoxic conditions mitigated the root growth inhibition induced by hypoxic stress in a concentration-dependent manner (Fig. 5a). The most alleviating effect was observed when 1 mM concentration of SNP was used, while higher SNP concentration did not result in a further increase of this alleviating effect. Similarly to SNP, hypoxia-evoked post-hypoxic root growth inhibition was alleviated by another NO donor, GSNO (Fig. 5b). However, GSNO had a similar effect as 1 mM SNP already at 0.2–0.4 mM concentrations. In addition, at higher GSNO concentration, we observed a strong decline of the GSNO-alleviating effect on the post-hypoxic root growth inhibition. Application of PFiC (which, similarly to SNP, releases cyanide and acts as an extracellular electron acceptor) during hypoxia slightly increased the root growth at 1–2 mM concentrations; however, there was high variability in individual root growth (Fig. 5c).

**Fig. 6:**
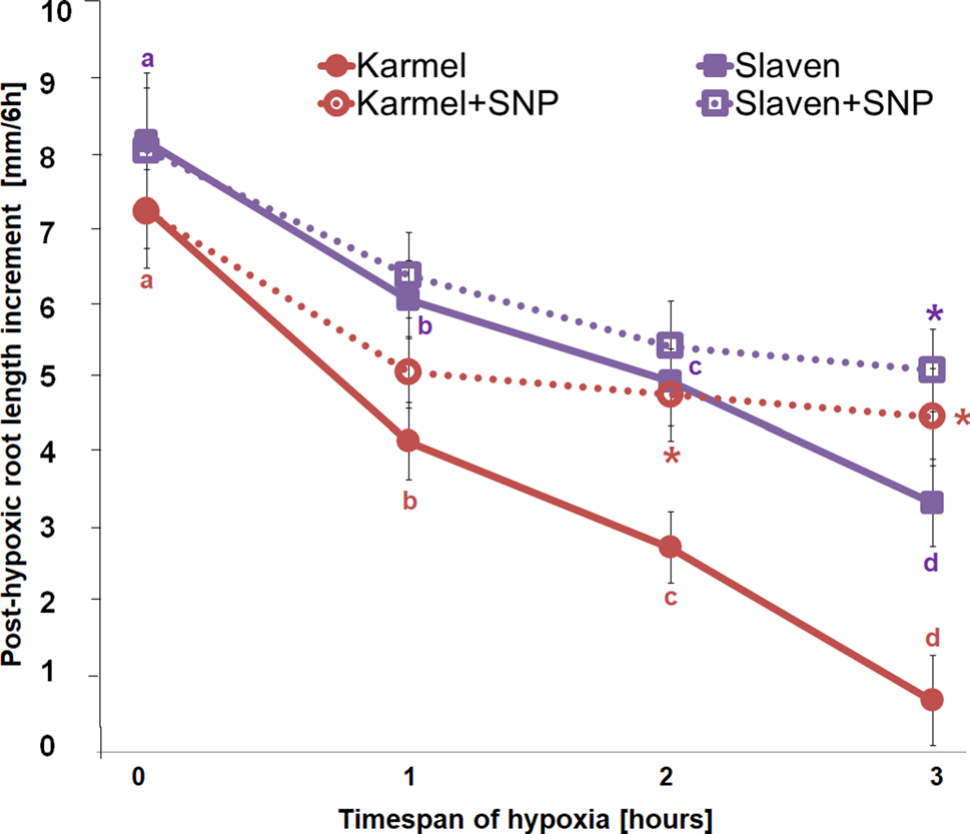
Root length increment during 6 h of recovery of barley cvs. Karmel and Slaven after 0 (normoxia), 1, 2 and 3 h of hypoxia without or with SNP treatment. Mean values ± SD (*n* = 5). Different letters and asterisk indicate statistical significance according to Tukey’s test (*P < 0.05*) between control and hypoxic stress or between hypoxic stress without and with SNP treatment, respectively

Root growth was inhibited within an hour during reoxygenation after hypoxic conditions in both cvs., and this inhibition of post-hypoxic root growth increased with the increasing time of the hypoxic stress conditions (Fig. 6). The sharpest decline in root growth was recorded for the cv. Karmel in comparison with cv. Slaven. Roots of the cv. Karmel, after 3 h of hypoxic stress, had almost no post-hypoxic root growth. In contrast to cv. Karmel, no root growth arrest but only severe post-hypoxic root growth inhibition was observed after 3 h of hypoxic stress in the cv. Slaven. While treatment of cv. Slaven with 1 mM SNP during hypoxic conditions only slightly increased the post-hypoxic root growth compared to roots without SNP treatment during hypoxic condition, in cv. Karmel, the post-hypoxic root growth was restored and reached the rate of cv. Slaven (Fig. 6).

**Fig. 7:**
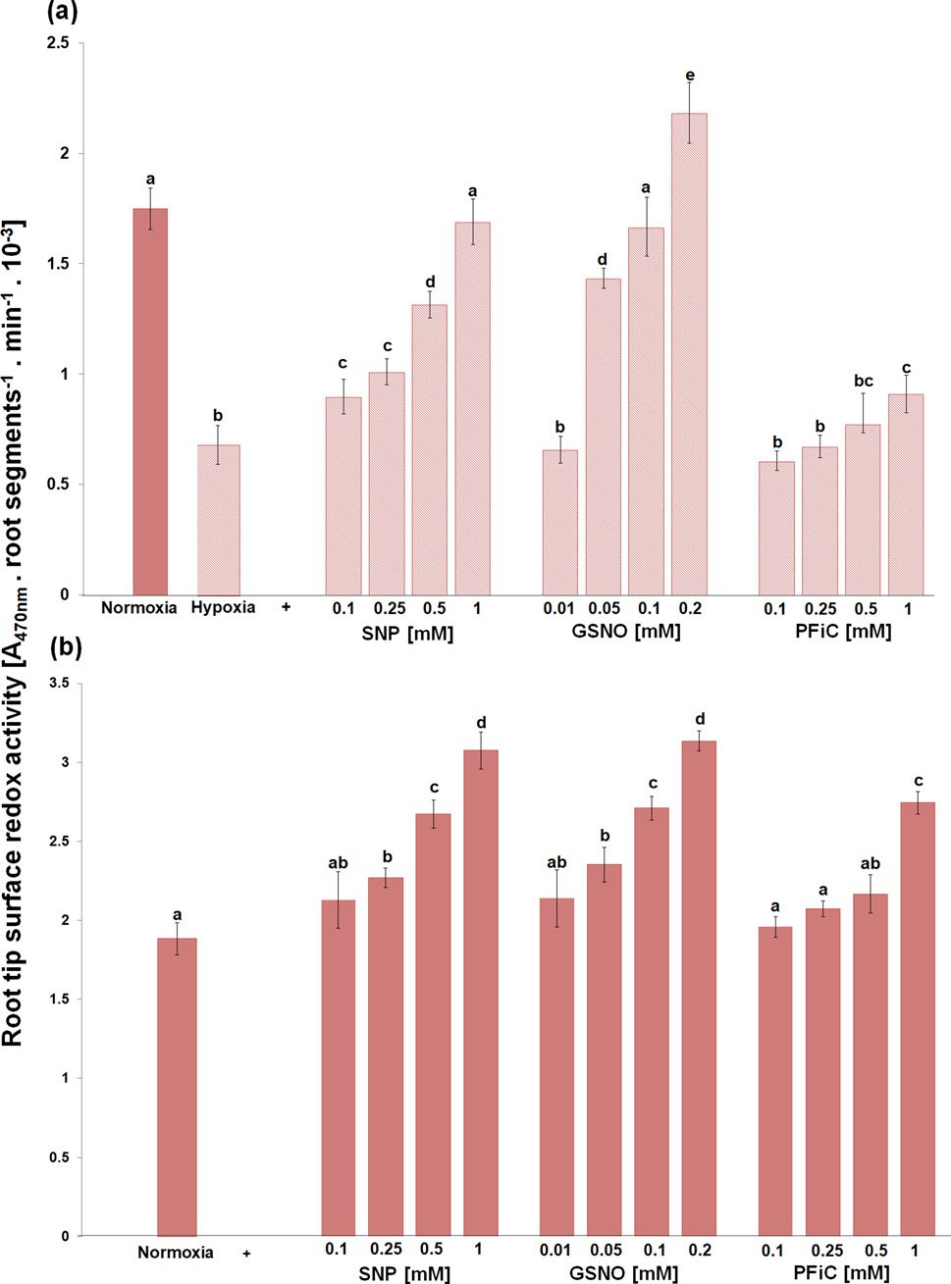
Effect of SNP, GSNO and PFiC during 3 h of hypoxia (**a**) and during short-term 30 min treatment of normoxic roots (**b**) on the root tip surface redox activity of barley cv. Karmel. Mean values ± SD (*n* = 5). Different letters indicate statistical significance according to Tukey’s test (*P < 0.05*)

**Fig. 8:**
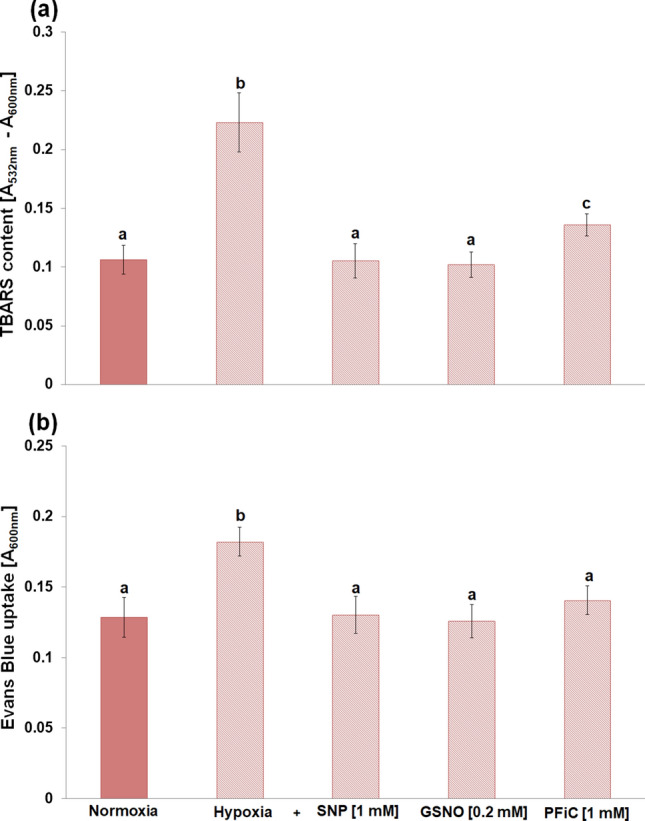
Effect of SNP, GSNO and PFiC during 3 h of hypoxia on the lipid peroxidation (**a**) and cell death (**b**) in the root tips of barley cv. Karmel. Mean values ± SD (*n* = 5). Different letters indicate statistical significance according to Tukey’s test (*P < 0.05*)

**Fig. 9:**
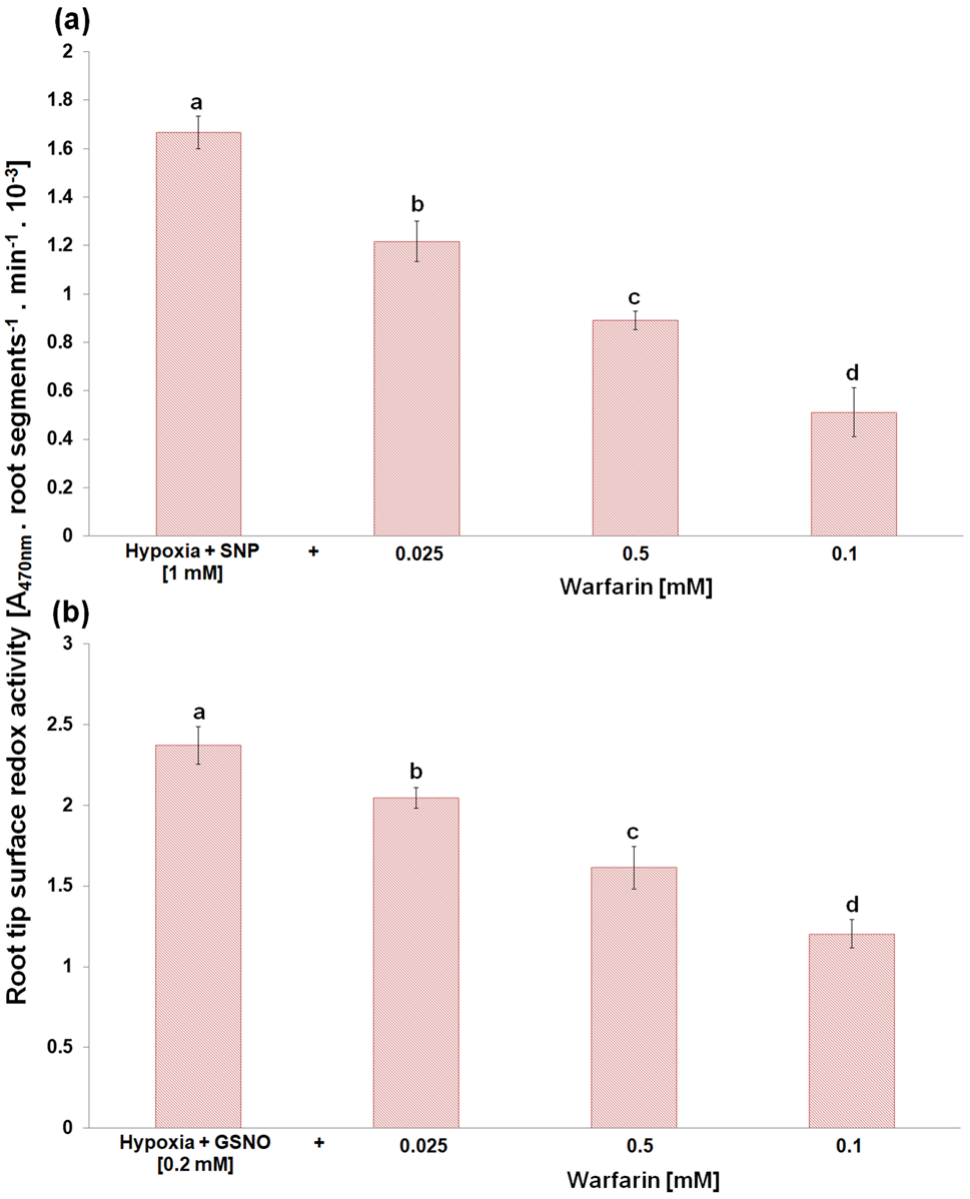
Effect of warfarin in combination with SNP (**a**) or GSNO (**b**) during 3 h of hypoxia on the root tip surface redox activity of barley cv. Karmel. Mean values ± SD (*n* = 5). Different letters indicate statistical significance according to Tukey’s test (*P < 0.05*)

Both NO donors, SNP and GSNO, significantly increased the surface redox activity of root tips under hypoxic conditions in a dose-dependent manner (Fig. 7a). While SNP at 1 mM, GSNO already at 0.1 mM concentration restored this redox activity of root tips to the level of control seedlings growing under normoxic conditions. Moreover, treatment with 0.2 mM GSNO caused higher root surface redox activity in comparison with control root tips. In turn, PFiC, as an extracellular electron acceptor, increased this redox activity to a much lower extent compared to SNP and GSNO. In addition, the application of SNP, GSNO and PFiC increased the surface redox activity of unstressed root tips even after 30 min of treatments (Fig. 7b). At the same time, besides the root surface activity restoration under hypoxic conditions, both SNP and GSNO completely eliminated hypoxic stress-induced lipid peroxidation in the root tips as well as cell death (Fig. 8). In turn, the SNP- or GSNO-caused improvement in redox activity during hypoxic stress was reversed by the addition of warfarin, an inhibitor of plasma membrane electron transport, in a concentration-dependent manner (Fig. 9).

## Discussion

Most crop plants are very sensitive to flooding; notably, barley is more sensitive than other cereals (Setter and Waters 2003). Flooding-induced hypoxia negatively affects all plant developmental stages, but the seedling establishment after germination is the most vulnerable period during plant growth. Even rice, a highly submergence-tolerant cereal crop, is more sensitive to hypoxic conditions during germination and early seedling establishment in comparison with later developmental stages (Kumar et al. 2020). Furthermore, in rapeseed seedlings, both root and shoot development is dramatically affected by hypoxic stress (Guo et al. 2020). In this study, we showed that the dissolved oxygen concentration in distilled water decreased rapidly after the immersion of roots in a seedling number- and time-dependent manner (Table 1). These results are in agreement with a previous study in which both cucumber and wheat root systems, due to cell respiration, reduced dissolved oxygen concentration in a nutrient solution without oxygen replenishment within an hour, resulting in a hypoxic (below 3 mg/L) condition (Morard and Silvestre 1996). In line with these observations, we present evidence that at the early seedling stage, even short-term hypoxic stress causes a strong inhibition of post-treatment root growth in both analyzed cvs. of barley (Fig. 6). Moreover, the root growth of cv. Karmel stopped completely when the roots were exposed to hypoxic conditions by the partial submergence of seedlings into distilled water for 3 h. On the contrary, in cv. Slaven, the post-hypoxic root growth was not fully inhibited; however, it was strongly reduced in comparison with control seedlings, indicating that cv. Karmel is more sensitive to sudden short-term hypoxic stress than cv. Slaven.

In cv. Karmel, the root growth arrest induced by the exposure of barley roots to hypoxic stress for 3 h was accompanied by a significant increase in lipid peroxidation and extensive cell death in the root tips (Fig. 1). In accordance with these results, it has been recently described that a marked increase in lipid peroxidation is characteristic of hypoxia-sensitive barley seedlings, but it is not the case for hypoxia-tolerant sea barley under waterlogging (Xu et al. 2022). Similarly, ROS accumulation in soybean roots and leaves during the first hours of waterlogging was followed by elevated levels of lipid peroxides (Da-Silva and do Amarante 2020a). A previous study has also shown that hypoxic conditions in submerged plant tissues cause cell damage, especially via lipid peroxidation (Santosa et al. 2007). In comparison with barley, in Arabidopsis seedlings, during hypoxia stress shorter than 4 h, root tips die mainly during the re-oxygenation phase**,** whereas longer hypoxia (4.5 h) results in meristem death in root tips during both the hypoxia and the subsequent re-oxygenation phase (Liu et al. 2022).

Despite the increase in lipid peroxide level, a considerably enhanced superoxide level was not detected in the hypoxia-stressed root tips when HE or NBT was used (Suppl. Fig. S2, S3). It has been reported that a non-charged membrane-permeable HE is rapidly taken up by the cells and cell organelles, after which, upon HE oxidation, the fluorescence is mainly associated with superoxide produced by mitochondria (Meany et al. 2006; Valentovičová et al. 2022). In our experiments, short-term hypoxic stress did not increase but rather slightly decreased the oxidation of HE in the root tips, indicating that mitochondrial superoxide in barley root tips is not responsible for hypoxia-induced lipid peroxidation. In our previous work, we showed that NBT, after the inhibition of oxidoreductases and peroxidases in intact roots, reacts preferentially with the superoxide generated by DPI-sensitive NADPH oxidases in the apoplast of the root elongation zone (Valentovičová et al. 2022). In the current experiments, hypoxic stress strongly inhibited this DPI-sensitive superoxide generation in the root elongation zone of cv. Karmel. In accordance with these results, it has been shown that in the barley roots of sensitive genotypes, a decrease in superoxide generation is a characteristic symptom of waterlogging, whereas increased superoxide generation was observed in waterlogging-tolerant genotypes in comparison with control plants (Luan et al. 2018a). It has been shown that hydrogen peroxide, which originated from superoxide generated by the NADPH oxidase, plays a crucial signaling role in the induction of hypoxia tolerance mechanisms (Yang and Hong 2015; Yamauchi et al. 2017).

Under long-term (several days) flooding stress, significant increases in the antioxidant enzyme activities of barley roots were detected in both hypoxia -tolerant and -sensitive genotypes (Luan et al. 2018b). On the contrary, GR activity was not altered in roots during the exposure of barley seedlings to anoxic conditions for 3 h (Skutnik and Rychter 2009). According to these results, short-term, a few hours long, hypoxic conditions did not cause significant changes in the activity of GPX and GR (Suppl. Fig. S4), which are responsible for the elimination of both hydrogen and lipid peroxides and for the regeneration of the reduced glutathione pool, respectively. However, it has previously been reported that peroxides, as substrates for GPX, are the main activators of GPX expression in plants exposed to abiotic stress (Avsian-Kretchmer et al. 1999). In agreement with this, in our experiments, peroxides detected by thiobarbituric acid were increased only 3 h after the hypoxic stress, therefore; their amount probably did not reach the required levels to increase GPX activity within short-term flooding-induced hypoxia.

In contrast to NBT, XTT, due to its net negative charge, is largely cell-impermeable and forms water-soluble formazan; furthermore, its reduction requires an intermediate electron acceptor. Therefore, this reduction probably occurs at the cell surface or at the level of the plasma membrane via trans-plasma membrane electron transport systems (Berridge et al. 2005). In this study, we observed a decrease in root tip surface redox activity in both cvs.. However, in the cv. Karmel, this decrease was more intensive than in cv. Slaven and was significant even after 1 h of hypoxic conditions (Fig. 4a). These results suggest that at the very early phase of hypoxic stress, the impairments in plasma membrane electron transport systems are probably responsible for the enhanced lipid peroxidation and cell death in the barley root tips. Numerous components of plant plasma membrane electron transport pathways have been implied to function in both ROS generation and scavenging (Lüthje et al. 2013). It has been previously shown that in HL-60 cells, the plasma membrane redox system has a key role in protecting the cells against lipid peroxidation and cell death induced by oxidative stress (Rodríguez-Aguilera et al. 2000). On the other hand, even a small disturbance in these electron transport systems, such as the removal of substrate or their inhibition, may lead to ROS generation (Vuletić et al. 2005; Biniek et al. 2017). The involvement of plasma membrane redox system-generated ROS in membrane damage due to enhanced lipid peroxidation has also been reported *in vitro* in the purified wheat roots plasma membrane (Qiu and Liang 1995). In the isolated plasma membrane, an oxygen-insensitive NADH-stimulated mechanism is generating the hydroxyl radical; this process is enhanced by the inhibition of superoxide-generating NADPH oxidase (Mojović et al. 2004).

In contrast to animal tissues, plant tissues made up of cells with a cell wall contain large amounts of peroxidases and oxidoreductases in their extracellular spaces. NO is a substrate for numerous of these enzymes, which considerably hamper the detection of extracellular NO released from root cells or generated directly in the extracellular spaces. It has previously been shown that NO is a general and physiological substrate for both animal and plant peroxidases (Glover et al. 1999; Abu-Soud and Hazen 2000). In addition, roots possess a high capacity for NO metabolism via NO-dioxygenase activity (Igamberdiev et al. 2004). Both peroxidases and dioxygenases are very sensitive to KCN; therefore, we analyzed the effect of KCN on the amount of DAF-FM-detectable NO released by the root tips into the incubation/reaction mixture (Suppl. Fig. S1). In our experimental conditions, the presence of KCN markedly increased the amount of DAF-FM-detectable extracellular NO, which increased with the increasing number of root tips in the reaction mixture, whereas without KCN, it was hardly detectable. In accordance with these results, in further experiments, we used 1 mM KCN and 15 root tips per reaction mixture for extracellular NO detection by DAF-FM.

A large increase in NO production has been previously reported in alfalfa root cultures grown under hypoxic conditions compared to aerobic conditions (Dordas et al. 2003). In our experiments, extracellular NO generation in the root tips was observed in the more tolerant cv. Slaven, while in cv. Karmel, characterized by increased lipid peroxidation and cell death, a change in NO level was not detected (Fig. 2a). In turn, we did not detect significant alterations in intracellular NO level during short-term flooding-induced hypoxia in the barley root tip (Fig. 3), suggesting its crucial role in the apoplast of barley root tips. The NO level was higher in hypoxia-tolerant rice in comparison with hypoxia-sensitive barley, suggesting that higher NO turnover results in better adaptation of rice seedlings to hypoxic conditions (Jayawardhane et al. 2021). Thus, we assume that this increased level of extracellular NO may be responsible for the maintenance of vital cell functions or for the activation of some tolerance mechanisms in the more hypoxia-tolerant cv. of barley. The application of NO donors, either SNP or GSNO, during the short-term flooding-induced hypoxic stress revealed a marked alleviating effect on the hypoxia-induced inhibition of root surface redox activity (Fig. 7), which was accompanied by a considerable reduction of lipid peroxidation and renewal of root growth (Fig. 5, 6 ,8). This very early production of NO under hypoxic conditions was also observed in the maize root tips (Mugnai et al. 2012). In addition, the application of NO donors markedly increased the survival rate of maize roots under hypoxia (Mugnai et al. 2012; Li et al. 2023) and mitigated the negative effect of short-term flooding stress in soybean (Khan et al. 2019). Similarly to the NO-induced alleviating effect on hypoxia-induced cell death in the barley root tip, the accumulation of ROS and cell death in pea roots were prevented by hydrogen sulfide, which can act as a crucial signaling molecule under hypoxic conditions (Cheng et al. 2013). However, it has been reported that for the induction of hydrogen sulfide, an enhanced NO level was responsible under hypoxia (Peng et al. 2016).

Increasing evidence indicates that the main source of NO in hypoxic plant cells is the mitochondrial reduction of nitrite to NO. In isolated pea mitochondria, the NO generation started and increased markedly within a few minutes under hypoxia (Gupta et al. 2017). Enhanced nitrate supply, via the enhanced NO level-mediated ROS decrease, markedly increased the waterlogging tolerance of soybean seedlings (Da-Silva and do Amarante 2020b). Moreover, nitrate nutrition-induced enhanced NO production intensified hypoxia tolerance in Arabidopsis due to the improved efficiency of the phytoglobin-NO cycle and fermentative pathways, increased alternative oxidase capacity and decreased ROS generation (Wany et al. 2019). Indeed, a very high activity of the phytoglobin-NO cycle under hypoxic conditions may lead to lower emission of NO from roots (Cochrane et al. 2017). In addition, NO is directly involved in the maintenance of mitochondrial integrity by decreasing ROS production and lipid peroxidation under low oxygen level (Gupta et al. 2017).

Some studies revealed that NO decreased the level of ROS through the activation of the cell antioxidant apparatus (Shi et al. 2005; Ahmad et al. 2018). However, in waterlogged soybean seedlings, nitrate supply-evoked enhanced NO level decreased the activity of antioxidant enzymes in comparison with seedlings without nitrate nutrition (Da-Silva and do Amarante 2020b). Authors suggested that NO itself may function as an antioxidant molecule, e.g. through scavenging superoxide. Rapid modification of the mitochondrial electron transport pathway by NO, such as inhibition of mitochondrial complex IV, can decrease oxygen consumption, while activation of alternative respiration by NO can reduce superoxide generation (Gupta et al. 2018). On the other hand, neuronal cells with impaired mitochondrial function up-regulated plasma membrane-associated redox system, which protects the cell from oxidative stress (Hyun et al. 2007). In barley roots (cv. Karmel), we did not observe an enhanced superoxide generation during the short-term hypoxic conditions, and we showed that high lipid peroxidation level occurred due to the inhibition of root redox activity, which was rapidly restored by the application of NO donors during the hypoxic conditions (Fig. 7). It has been previously shown that NO reacts rapidly not only with superoxide but also with alkoxy or peroxy radicals; therefore, NO may be involved directly in limiting membrane damages by terminating the lipid peroxidation chain reaction (Wink and Mitchell 1998). Meanwhile, in our experiments, the vitamin K antagonist warfarin, an inhibitor of the plasma membrane electron transport system (Döring et al. 1992), mitigated the positive effect of SNP or GSNO on the root tip surface redox activity in a concentration-dependent manner (Fig. 9). In addition, under hypoxic conditions NO was detectable mainly in the extracellular spaces with the cell impermeable fluorescent probe, while no significant changes in NO level were observed inside the root cells when the cell permeable probe was used. These results indicate that NO is probably involved in the maintenance of root surface redox activity under hypoxic stress.

To evoke NO depletion in root tips and to study its subsequent effect on the root surface redox activity, the root tips were treated with the NO scavenger cPTIO (Fig. 4b). We showed that similarly to cPTIO-evoked extracellular NO depletion, high (0.5–1 mM) cPTIO concentrations were required for the inhibition of the root tip surface redox activity. The requirement of these higher cPTIO concentrations and the ineffectiveness of cPTIO on intracellular NO depletion can be attributed to the low efficiency of cPTIO as an NO scavenger in plant cell cultures and intact seedlings due to its cell impermeability, slow uptake, relative instability, and mainly due to its rapid degradation in the presence of cells with high metabolic activity (Planchet and Kaiser 2006; D´Alessandro et al. 2013). Nevertheless, this opposite effect of NO scavenger on the root surface activity in comparison with NO donors further supports the role of NO in the maintenance of transmembrane redox activity in root tips.

## Conclusion

A rapid onset of hypoxic stress can occur in barley root tips within hours of root exposure to flooding. The cv. Karmel proved to be more susceptible to sudden hypoxic stress than cv. Slaven. Whereas this sudden flooding-induced hypoxic stress triggers root growth arrest accompanied by increased lipid peroxidation and cell death within 3 h in cv. Karmel, in the more tolerant cv. Slaven, it causes only reduced root growth rate, while the extracellular NO level increases in the root tips. The root tip surface redox activity decreases with the increasing timespan of hypoxia in both cvs.. However, this decrease in redox activity started earlier and was greater in the cv. Karmel in comparison with cv. Slaven. In the more hypoxia-sensitive cv. Karmel, the reduction of the root tip surface redox activity was evident within 1 h of hypoxic conditions suggesting that the alterations in the plasma membrane electron transport system are the very early symptom of barley root tip under hypoxic conditions. These differences between the cultivars during hypoxia could be attributed to the observed changes in NO levels, as the hypoxic conditions in cv. Slaven are associated with elevated extracellular NO level in the root tips. Furthermore, the application of NO donors to cv. Karmel increased its hypoxia tolerance, suggesting that extracellular NO plays a key role in maintaining the root tip surface redox activity and in the restriction of lipid peroxidation and cell death under short-term hypoxic stress in the root tips of barley seedlings.

### Supplementary Information

Below is the link to the electronic supplementary material.

Supplementary file1 (TIF 5530 KB)

Supplementary file2 (TIF 69158 KB)

Supplementary file3 (TIF 3657 KB)

Supplementary file4 (TIF 48344 KB)

Supplementary file5 (TIF 3598 KB)

Supplementary file6 (TIF 6918 KB)

## Data Availability

All data generated or analyzed during this study are included in this published article

## Abbreviations

cPTIO: 2-(4-carboxyphenyl)-4,4,5,5-tetramethylimidazoline-1-oxyl-3-oxide
DPI: Diphenyleneiodonium
GPX: Glutathione peroxidase
GR: Glutathione reductase
GSNO: S-nitroso-L-glutathione
HE: Hydroethidine
NBT: Nitro-blue tetrazolium chloride
PFiC: Potassium ferricyanide
SNP: Sodium nitroprusside
