# Supplementary figures and images for: Extracellular nitric oxide sustains root surface redox activity and growth under sudden flooding-induced hypoxic conditions in barley root tips
Source: Planta. 2023 Nov 21;259(1):3. doi: 10.1007/s00425-023-04279-w (PMC10663193; doi:10.1007/s00425-023-04279-w)

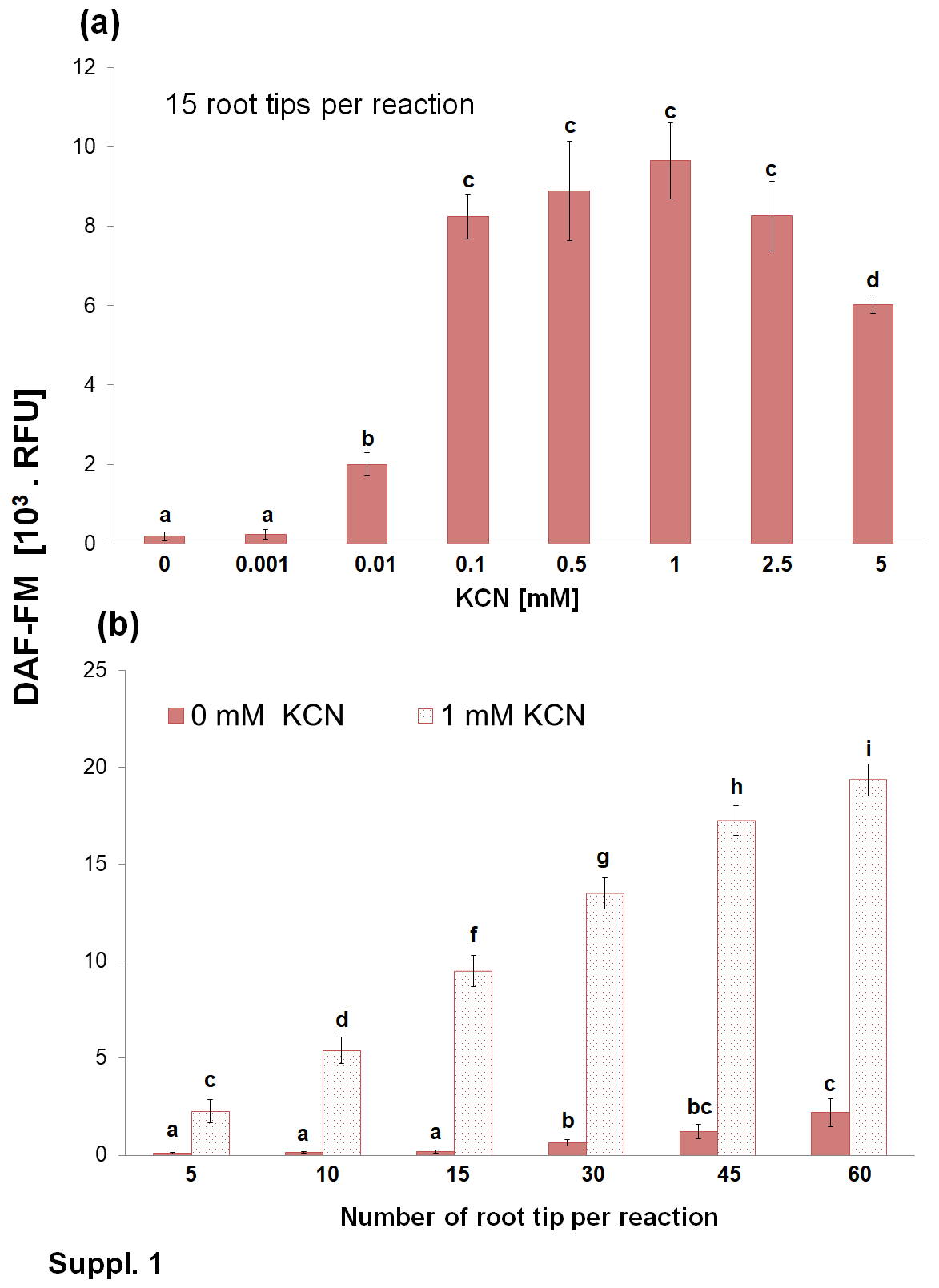

Supplement: Supplementary file 1 — Supplementary file1 (TIF 5530 KB) [file 425_2023_4279_MOESM1_ESM.tif]

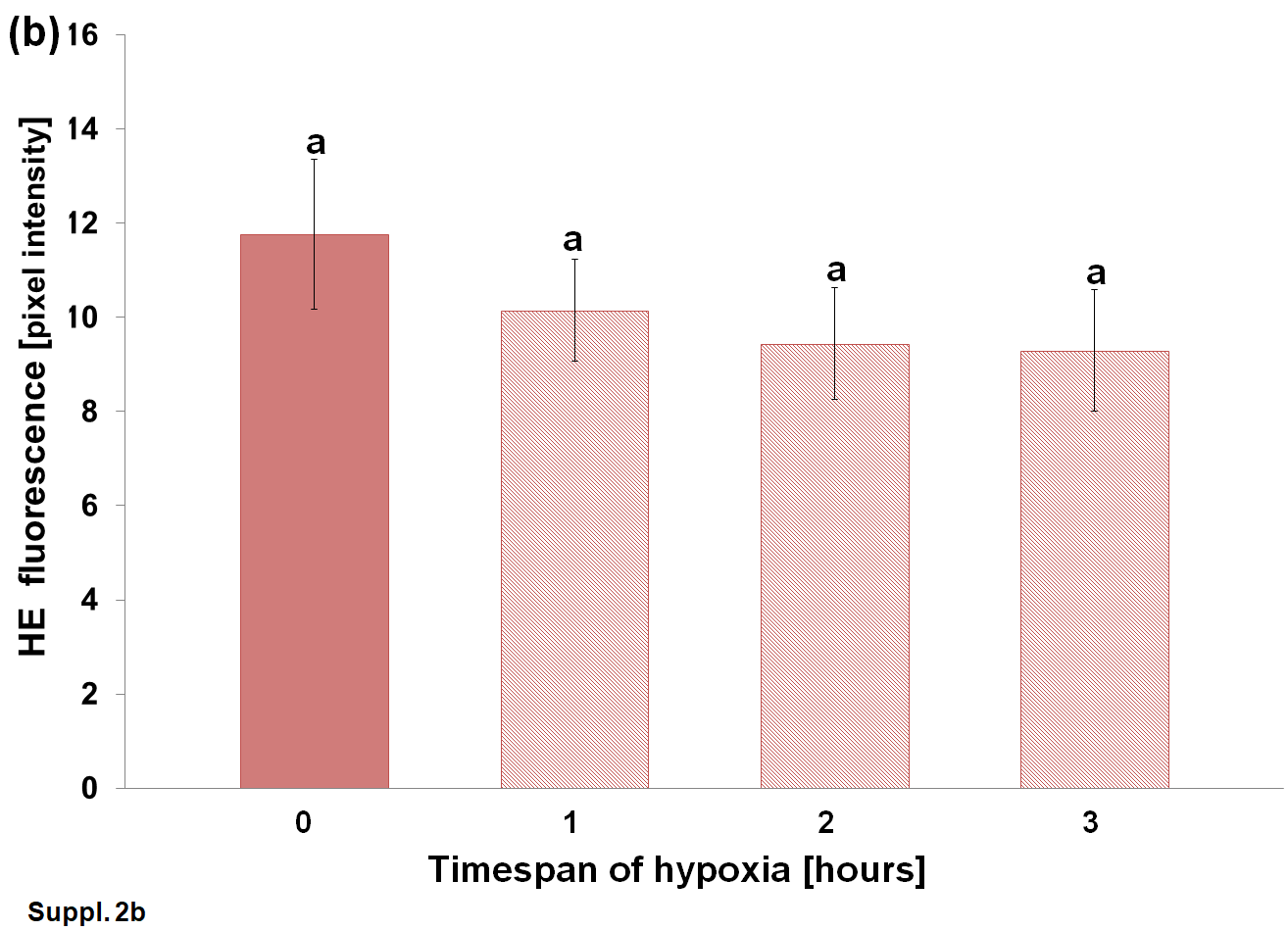

Supplement: Supplementary file 3 — Supplementary file3 (TIF 3657 KB) [file 425_2023_4279_MOESM3_ESM.tif]

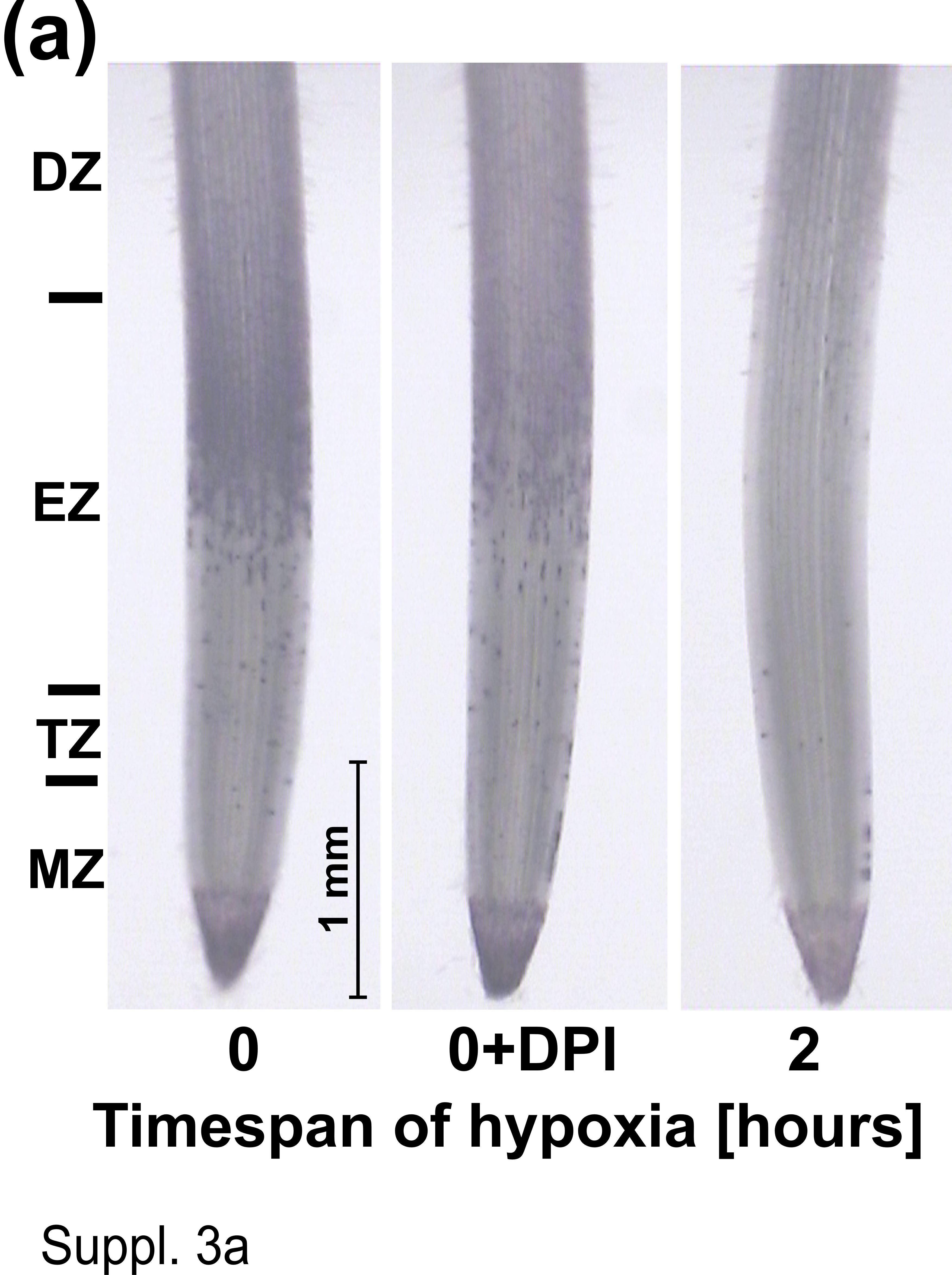

Supplement: Supplementary file 4 — Supplementary file4 (TIF 48344 KB) [file 425_2023_4279_MOESM4_ESM.tif]

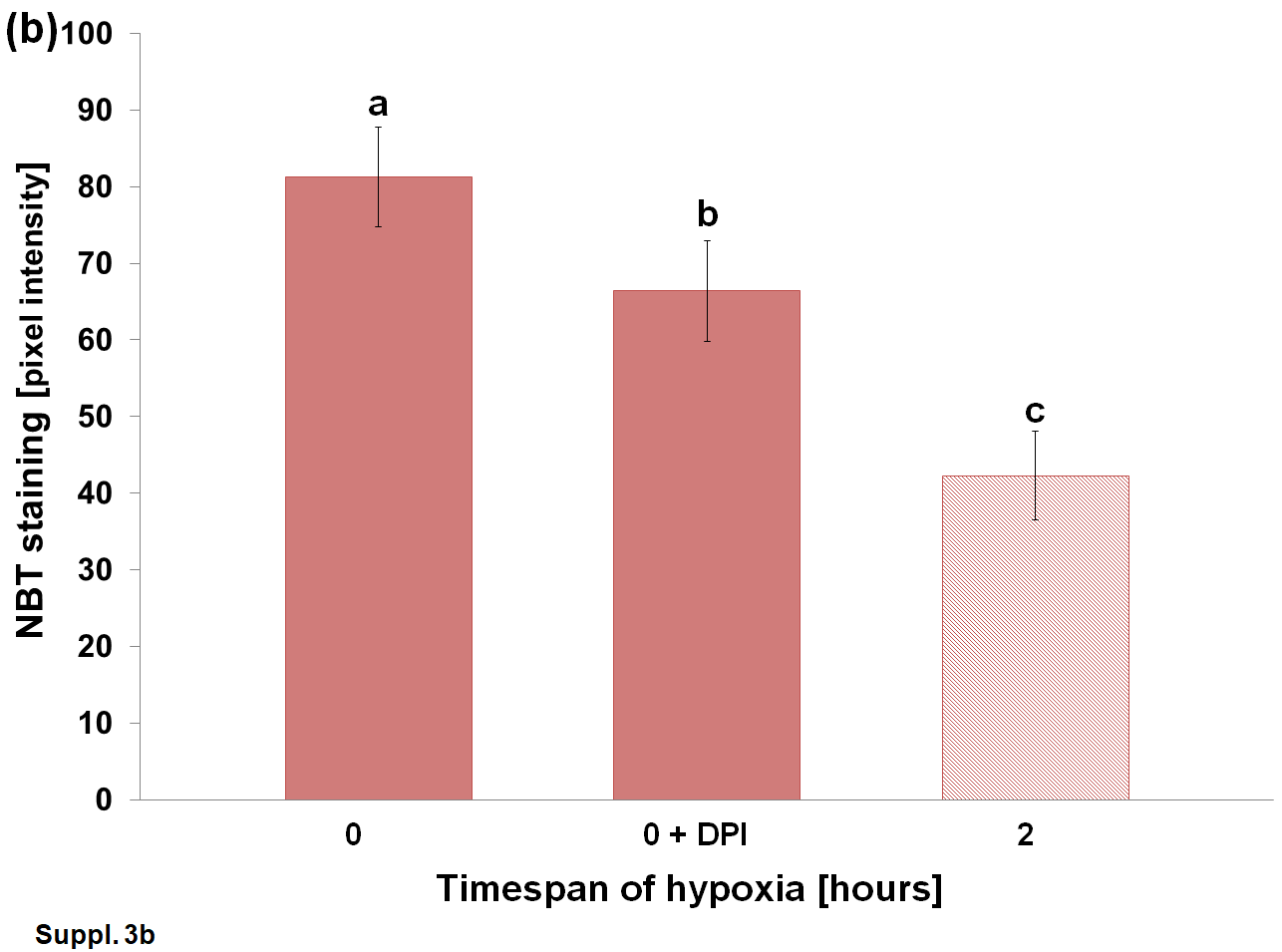

Supplement: Supplementary file 5 — Supplementary file5 (TIF 3598 KB) [file 425_2023_4279_MOESM5_ESM.tif]

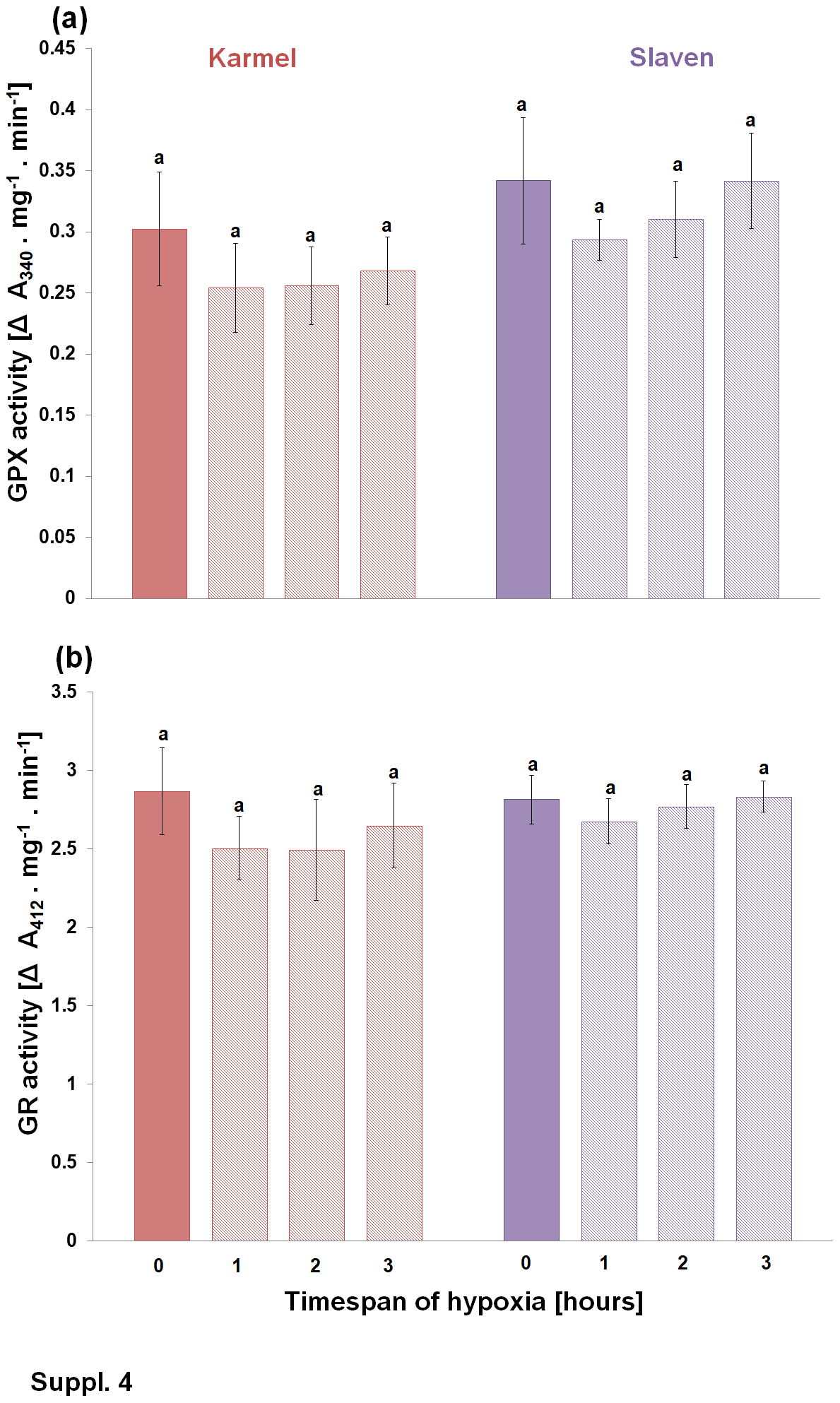

Supplement: Supplementary file 6 — Supplementary file6 (TIF 6918 KB) [file 425_2023_4279_MOESM6_ESM.tif]
